# Exogenous Spermidine Induces Cadmium Stress Tolerance in Cucumber Seedlings by Promoting Plant Growth and Defense System

**DOI:** 10.3390/toxics13100822

**Published:** 2025-09-26

**Authors:** Guangchao Yu, Ming Wei, Zhipeng Wang, Lian Jia, Yue Qu

**Affiliations:** 1 College of Chemistry and Life Sciences, Anshan Normal University, Anshan 114007, China; wm050820@163.com (M.W.); wangzp1326@gmail.com (Z.W.); jl_58@163.com (L.J.); quyue199209@163.com (Y.Q.); 2 Liaoning Key Laboratory of Development and Utilization for Natural Products Active Molecules, Anshan Normal University, Anshan 114007, China

**Keywords:** cucumber, spermidine (Spd), cadmium (Cd) stress, physiologic index, cadmium content, genes expression

## Abstract

This study aims to investigate the role of exogenous spermidine (Spd) in mitigating the adverse effects of cadmium (Cd) stress on the growth and development of cucumber (*Cucumis sativus*). The cucumber cultivar “Xintaimici” was used as the experimental material, and a hydroponic experiment was carried out. Based on a baseline Cd concentration of 10 mg·L^−1^, Spd was supplemented at concentrations of 0.05, 0.1, 0.2, 0.4, and 0.5 mM, resulting in seven treatment groups: control group (CK), S0 group (Cd-only treatment, 10 mg·L^−1^ Cd + 0 mM Spd), S1+ Cd group (10 mg·L^−1^ Cd + 0.05 mM Spd), S2+ Cd group (10 mg·L^−1^ Cd + 0.1 mM Spd), S3+ Cd group (10 mg·L^−1^ Cd + 0.2 mM Spd), S4+ Cd group (10 mg·L^−1^ Cd + 0.4 mM Spd), and S5+ Cd group (10 mg·L^−1^ Cd + 0.5 mM Spd). This study analyzed the regulatory effects of Spd on the growth and development, antioxidant capacity and cadmium accumulation characteristics of cucumber seeds and seedlings. It was found that cadmium stress significantly inhibited their growth process and led to a decline in multiple physiological indicators. Under a Cd concentration of 10 mg·L^−1^, the application of 0.2 mM Spd significantly improved these parameters. During the seedling stage, the application of 0.2 mM Spd under Cd stress significantly enhanced the activities of superoxide dismutase (SOD), peroxidase (POD), catalase (CAT), and ascorbate peroxidase (APX), as well as the content of soluble proteins, while significantly reducing malondialdehyde (MDA) levels. Cd content analysis revealed that 0.2 mM Spd promoted Cd accumulation in roots while suppressing its translocation to young leaves, thereby reducing Cd accumulation in aboveground tissues. Gene expression analysis demonstrated that this treatment significantly upregulated the expression levels of the phytochelatin synthase gene (*CsPCS1*) and the gene associated with reduced glutathione synthesis (*CsGSHS*). In conclusion, the exogenous application of 0.2 mM Spd effectively alleviates oxidative damage and osmotic stress induced by Cd stress in cucumber, promotes plant growth, and significantly enhances Cd tolerance.

## 1. Introduction

The rapid advancement of science and technology has significantly propelled societal progress in both industrial and agricultural sectors; however, it has concurrently exacerbated the issue of heavy metal contamination in soils. Major sources of heavy metal pollution include the excessive and improper application of fertilizers and pesticides, mining activities, metallurgical processing, emissions from fossil fuel combustion (“three wastes”), and the use of wastewater containing heavy metals for agricultural irrigation [1]. Cadmium (Cd) is a non-essential heavy metal that poses severe threats to plant growth and development. Cd undergoes rapid uptake by plant roots and is transported to the aerial parts via the xylem [2]. Upon reaching a threshold concentration within plant tissues, Cd induces toxic effects that hinder physiological processes [3]. Typically, Cd accumulates predominantly in roots, rendering them one of the most directly and severely affected organs [4]. Although above-ground tissues such as leaves are more sensitive to Cd toxicity, the translocation of Cd from roots to shoots still exerts significant phytotoxic effects. When Cd concentrations surpass safe thresholds, plants exhibit visible symptoms of damage, including chlorosis and abscission of leaves, stunted growth, delayed developmental stages, reduced biomass, and, in severe cases, mortality [5].

Currently, physical and chemical remediation techniques are the primary approaches for mitigating environmental Cd pollution. However, these methods are often associated with high costs and the risk of secondary contamination. In contrast, phytoremediation technology, as a novel and environmentally sustainable approach for mitigating soil cadmium contamination, has shown considerable potential for application in the remediation of contaminated sites in recent years, owing to its high sustainability, broad applicability, and minimal environmental impact. Nevertheless, plant tolerance to heavy metals tends to decline as Cd concentration increases or exposure duration extends. For instance, Huang et al. [6] reported that a 10 μM Cd treatment significantly reduced root length, root surface area, and root tip number in pepper plants, indicating substantial inhibition of root growth. Similarly, increasing Cd concentrations progressively impaired the growth and development of tobacco seedlings (*Nicotiana tabacum* L.), with a 73.1% reduction in biomass observed under 40 μM Cd treatment compared to the control [7]. Notably, a concentration-dependent relationship between Cd stress and plant growth has been identified. At low concentrations, Cd may exert mild stimulatory effects on plant growth. However, as exposure concentration and duration increase, toxic effects become increasingly pronounced [8]. For example, treatment with 10 mg·L^−1^ Cd slightly enhanced the germination rate of Rumex acetosa and the vigor of Mimosa pudica. However, further increases in Cd concentration led to a decline in germination rate, germination potential, and vigor index in riparian herbaceous species [9].

In recent years, the investigation of plant tolerance mechanisms against heavy metal stress has emerged as a focal area within environmental science. In this context, exogenous application of regulatory substances has garnered increasing scientific attention due to its distinct advantages, such as operational simplicity, notable enhancement of stress resistance, and environmental compatibility [10]. This approach has become a crucial strategy for improving plant tolerance to heavy metals. Polyamines (PAs), a group of biologically active low-molecular-weight aliphatic nitrogen-containing compounds containing two or more amino groups, serve as essential metabolic regulators in plants. These signaling molecules play a critical regulatory role in diverse physiological processes, including fundamental functions such as cell division, embryogenesis, root morphogenesis, and seed germination, as well as the biosynthesis and signal transduction of plant hormones and various abiotic stress response mechanisms [11,12]. Owing to their significant regulatory functions in plant physiology, some researchers have proposed that PAs may function as a novel class of plant hormones. However, notable differences exist between PAs and classical plant hormones in terms of effective concentration and transport mechanisms. Consequently, alternative perspectives classify PAs as signaling mediators in hormonal pathways, acting as second messengers akin to cyclic adenosine monophosphate (cAMP), or as regulatory factors influencing plant growth and development. Based on the number of amino groups present in their molecular structures, PAs are categorized into diamines, triamines, and tetramines. Among these, spermidine (Spd), the most ubiquitously distributed triamine in plants, has been recognized as a key exogenous regulator in enhancing crop resistance to abiotic stress [13,14,15]. Extensive studies have demonstrated that PAs can significantly improve plant resilience under abiotic stress conditions. Under heavy metal stress, exogenous application of Spd exerts protective effects through multiple mechanisms, including the activation of antioxidant enzymes, regulation of macromolecular metabolism, modulation of phytosiderophores (PSs), and iron metabolism-related enzymatic systems [16]. These mechanisms collectively contribute to the mitigation of heavy metal toxicity and influence the uptake and accumulation behavior of heavy metals in plants.

Numerous studies have investigated the effects of exogenous spermidine (Spd) on the physiological tolerance mechanisms of woody, herbaceous, and crop plants under heavy metal stress. Tang et al. [17] demonstrated that Spd application significantly elevated spermidine levels, soluble protein content, superoxide dismutase (SOD) activity, reduced ascorbic acid (ASA) concentration, glutathione reductase (GR) activity, and glutathione peroxidase (GPX) activity in *Salix matsudana* leaves. This treatment also reduced the accumulation of superoxide anion (O_2_^−^), hydrogen peroxide (H_2_O_2_), and malondialdehyde (MDA), thereby alleviating cadmium-induced oxidative stress. Gong et al. [18] reported that the protective effects of Spd on *Boehmeria nivea* (L.) Gaudich. under Cd stress became evident only after the toxic damage reached a certain threshold. Gu et al. [19] found that exogenous Spd enhanced the tolerance of rice seedlings to combined Cd and Pb stress, as evidenced by increased plant height, root length, and fresh and dry weights of roots and shoots. Furthermore, Spd application under Cd + Pb stress significantly reduced the levels of H_2_O_2_, O_2_^−^, and MDA, as well as the accumulation of Cd and Pb in plant tissues. Simultaneously, it increased the contents of mineral nutrients, carotenoids, chlorophyll, proline, soluble sugars, soluble proteins, total phenolics, flavonoids, and anthocyanins, along with antioxidant enzyme activities in both roots and shoots. Notably, 0.5 mM Spd exhibited the most pronounced protective effects against Cd- and Pb-induced physiological and metabolic impairments in rice seedlings. Additionally, the combined application of paclobutrazol and Spd has been shown to enhance cadmium tolerance in mung beans by up-regulating antioxidant and osmotic regulatory mechanisms [20].

Exogenous application of spermidine (Spd) has been demonstrated to enhance the antioxidant enzyme system in water chestnut leaves under copper stress, including ascorbate peroxidase (APX) and glutathione reductase (GR) [21]. When plants are subjected to cadmium stress, they activate a range of defense mechanisms to mitigate the associated cellular damage. These mechanisms are regulated at the molecular level by specific genes and can significantly elevate the levels of phytochelatins (PCs) in plant tissues [22]. Phytochelatins are a class of low-molecular-weight peptides synthesized from reduced glutathione (GSH) under the catalytic action of phytochelatin synthase (PCS). Under cadmium stress, PCS is activated, which promotes the synthesis of substantial amounts of PCs. These peptides subsequently bind with cadmium ions to form stable PC-Cd chelates [22]. The resulting chelates can be transported into the vacuole via ATP-binding cassette (ABC) transporters, thereby facilitating the compartmentalization and sequestration of cadmium. This process effectively reduces the concentration of free cadmium ions in the cytoplasm and alleviates their toxic effects [23]. Notably, existing studies have indicated that overexpression of the PCS gene does not consistently enhance plant tolerance to cadmium; in some cases, it may even increase sensitivity. This phenomenon may be attributed to the fact that PCs are not directly translated gene products but are synthesized through a dynamic equilibrium involving GSH. Selective overexpression of the PCS gene may lead to an increase in PC levels accompanied by a rapid depletion of GSH, thereby disrupting the homeostasis between their synthesis and degradation, which can result in metabolic disturbances within the plant [24]. Therefore, coordinated regulation of both PCS and GSH-related genes is essential for maintaining the functional integrity of the phytochelatin metabolic pathway.

To date, research on the application of exogenous Spd in vegetable crops has primarily focused on drought and salt stress tolerance, with limited studies addressing its physiological effects under Cd stress in cucumber seedlings. Therefore, this study aims to explore the potential of exogenous Spd as an effective strategy to enhance plant stress resistance and mitigate Cd toxicity in vegetable crops. Using cucumber seeds and seedlings as experimental materials, a hydroponic approach was employed to investigate the physiological and biochemical responses of cucumber plants under Cd-contaminated conditions supplemented with Spd. The study further examines the alleviating effects and underlying mechanisms of Spd on cucumber growth, providing a scientific basis for the management of Cd pollution in cucumber cultivation.

## 2. Materials and Methods

### 2.1. Plant Materials

The cucumber cultivar “Xintaimici” was used as the experimental material. All plants were cultivated in a controlled-environment soil culture facility at Anshan Normal University. Plump cucumber seeds were selected for the experiment and surface-sterilized prior to experimental treatment. A 120 mm diameter glass Petri dish was lined with two layers of filter paper as a germination substrate, and the seeds were incubated at a constant temperature of 28 °C for 24 h. Uniformly germinated seeds were then selected from the seedling tray and transferred to the Petri dishes. Each dish was lined with two layers of filter paper and loaded with 60 seeds. For the determination of germination and growth indicators of cucumber seeds.

In addition, cucumber seeds with consistent germination were selected and transplanted into hydroponic boxes. The plants were further cultivated with 1/2 Hoagland nutrient solution, and the nutrient solution was replaced once a week. The indoor conditions of the greenhouse were as follows: day temperature (24 ± 2) °C, night temperature (19 ± 2) °C, humidity 65–80%, light intensity 5000–8000 lx, and light duration 12 h·d^−1^.

### 2.2. Spd Concentration Screening

The preliminary experimental results demonstrated that under single cadmium ion stress, a cadmium concentration of 10 mg·L^−1^ exerted the most pronounced inhibitory effect on both cucumber seed germination and seedling growth. Therefore, this concentration was selected as the treatment condition for subsequent stress experiments.

Spermidine (Spd, analytical grade) was purchased from Aladdin Bio-Chem Technology Co., Ltd. (Shanghai, China). It was dissolved and prepared as a 200 mM stock solution, which was aliquoted and stored at −20 °C. Prior to experimental treatment, the stock solution was diluted to the required working concentrations. Preliminary experiments conducted by the research group demonstrated that to investigate the effects of Spd at varying concentrations on cucumber seed germination and seedling growth, three concentration levels—low, medium, and high—were established as treatment groups. In comparison with the control group, the 0.01 mM Spd treatment did not produce a statistically significant effect on seed growth. Based on these findings, this study selected 0.05, 0.1, 0.2, 0.4, and 0.5 mM Spd as the exogenous application concentrations for subsequent experimental analyses. Seven treatment groups were set up in this experiment, each consisting of three biological replicates, with each replicate comprising five cucumber plants. The names of the treatment groups are as follows: S0 (cadmium stress, 10 mg·L^−1^ Cd + 0 mM Spd), S1 + Cd (10 mg·L^−1^ Cd + 0.05 mM Spd), S2 + Cd (10 mg·L^−1^ Cd + 0.1 mM Spd), S3 + Cd (10 mg·L^−1^ Cd + 0.2 mM Spd), S4 + Cd (10 mg·L^−1^ Cd + 0.4 mM Spd), and S5 + Cd (10 mg·L^−1^ Cd + 0.5 mM Spd). A total of seven treatments were conducted.

### 2.3. Hydroponic Experiment

Following the initial placement of cucumber seedlings into the culture solution, a two-week acclimation period was implemented. Subsequently, the seedlings were transferred to hydroponic solutions containing cadmium and varying concentrations of spermidine (Spd) for experimental treatment. Cucumber seedlings cultivated in the complete nutrient solution without cadmium exposure served as the control group (CK). The treatment duration was set to 20 days, during which the hydroponic solution was renewed every 5 days, for a total of three solution changes. On the 20th day of treatment, the uppermost 1–2 fully expanded leaves were harvested from each seedling. One portion of the collected samples was immediately wrapped in aluminum foil, rapidly frozen in liquid nitrogen, and stored at –80 °C for subsequent biochemical analyses. Another portion of the samples was dried, ground into a fine powder, and used for the subsequent determination of cadmium content. The experimental treatments were as follows: CK, the control group, received no cadmium and was irrigated with an equivalent volume of distilled water. S0, the cadmium stress group, was cultivated in a nutrient solution containing 10 mg·L^−1^ Cd^2+^ and irrigated with distilled water. Groups S1 + Cd, S2 + Cd, S3 + Cd, S4 + Cd, and S5 + Cd were cultivated in nutrient solutions containing the same cadmium concentration (10 mg·L^−1^ Cd^2+^) and simultaneously treated with 0.05 mM, 0.1 mM, 0.2 mM, 0.4 mM, and 0.5 mM Spd solutions, respectively. In parallel, uniformly germinated cucumber seeds were subjected to the respective treatment solutions, and their growth and physiological parameters were assessed on the 7th day post-treatment.

### 2.4. Assessment of Germination Rate and Germination Potential

The number of cucumber seeds exhibiting effective germination was recorded after 3 and 7 days of incubation to calculate germination potential, germination rate, and germination inhibition rate. Effective germination was defined as the visible emergence of the embryonic radicle from the seed coat. Germination potential (%) = Number of normally germinated seeds on the 3rd day/Number of tested seeds × 100%; Germination rate (%) = Number of normally germinated seeds on the 7th day/Number of tested seeds × 100% [25].

### 2.5. Measurement of Plant Growth Parameters

Root Length and Hypocotyl Length: Following a 7-day incubation period, ten cucumber seedlings exhibiting uniform growth status were randomly selected from each treatment group. The lengths of the primary roots and hypocotyls were measured using a digital vernier caliper [26].

Fresh Weight and Dry Weight of Cucumber Seeds: The fresh weight of cucumber seeds was determined following 7 days of treatment. Subsequently, the samples were transferred to an oven, initially subjected to a 30 min blanching step at 105 °C to halt enzymatic activity, followed by drying at 80 °C for 72 h until a constant weight was achieved, after which the dry weight was recorded.

### 2.6. Determination of Physiological and Biochemical Indicators

Crude enzyme extraction: Accurately weigh 0.5 g of cucumber tissue and homogenize it in 2 mL of pre-cooled 50 mmol·L^−1^ phosphate buffer (pH 7.0) on ice. Transfer the homogenate to a centrifuge tube and rinse the residual material from the mortar with an additional 3 mL of pre-cooled phosphate buffer to ensure complete transfer. Centrifuge the suspension at 10,000× *g* rpm at 4 °C for 10 min. Carefully collect the supernatant for subsequent use as the crude enzyme extract.

The determination of superoxide dismutase (SOD) activity was conducted using the methods of Han et al. [27] and Liu et al. [28] with appropriate modifications. The total volume of the reaction system was 1.8 mL, which included: 0.3 mL of 0.026 mol·L^−1^ methionine (Met)-sodium phosphate buffer, 0.3 mL of 75 μmol·L^−1^ nitroblue tetrazolium (NBT), 0.3 mL of 20 μmol·L^−1^ riboflavin, 0.05 mL of crude enzyme extract, and 0.85 mL of distilled water. After thoroughly mixing the reaction mixture, it was divided into the test group and the control group: the test group was added with the crude enzyme extract, while the control group was replaced with an equal volume of phosphate buffer instead of the crude enzyme extract; both groups were then exposed to 4000 Lux light for 20 min and immediately stored in the dark. A blank group was also set up, without adding the crude enzyme extract and kept in the dark throughout the process, for zero calibration. After the reaction, the absorbance of each tube was measured at a wavelength of 560 nm. The activity unit of SOD was defined as the amount of enzyme required to inhibit the photoreduction of NBT by 50%, denoted as one unit (U). The calculation formula is as follows: SOD activity (U·g^−1^·min^−1^) = [(Ack − Ae) × V]/(Ack × FW × Vt × 0.5), where Ack represents the absorbance of the maximum photoreduction control tube, Ae represents the absorbance of the sample tube, V represents the total volume of the crude enzyme extract (mL), Vt represents the volume of the enzyme used in the test (mL), and FW represents the fresh weight of the sample (g).

The peroxidase (POD) activity was determined according to the methods of Han et al. [27] and Liu et al. [28] with appropriate modifications. The final volume of the reaction system was 1 mL, which contains 50 mmol·L^−1^ phosphate buffer (pH 6.0), 28 μL guaiacol and 19 μL 30% (*w*/*v*) H_2_O_2_. After thorough mixing, 20 μL of crude enzyme extract was added to initiate the reaction, and the reaction was immediately monitored by measuring absorbance at 470 nm at 1-min intervals for a total of three readings. The amount of enzyme required to cause an increase of 0.01 in absorbance per minute was defined as one unit (U) of peroxidase (POD) activity. The specific activity of POD was calculated using the following formula: POD activity (U·g^−1^·min^−1^) = (ΔA_470_ × Vt)/(0.01 × FW × Vs × T), where ΔA_470_ represents the linear rate of change in absorbance at 470 nm, T is the reaction time (min), Vt is the total volume of crude enzyme extract (mL), Vs is the volume of enzyme extract used in the assay (mL), and FW is the fresh weight of the sample (g).

The activity of catalase (CAT) was determined according to the method of Aebi [29] with appropriate modifications. The reaction system contained 2.9 mL of 50 mmol·L^−1^ phosphate buffer (pH 7.0) and 0.05 mL of crude enzyme solution. After thorough mixing, the mixture was pre-incubated in a water bath at 25 °C for 5 min. Then, 0.05 mL of 750 mmol·L^−1^ H_2_O_2_ was added to initiate the reaction. Immediately, the reaction mixture was divided into the test group and the control group: the test group contained the crude enzyme solution, while the control group was replaced with an equal volume of phosphate buffer. Absorbance was measured at 240 nm every 1 min, and three consecutive readings were recorded. One unit of enzyme activity was defined as an increase of 0.01 in the absorbance change per minute (ΔA_240_). CAT activity was calculated using the following formula: CAT activity (U·g^−1^·min^−1^) = (ΔA_240_ × Vt)/(0.01 × FW × Vs × T), where ΔA_240_ represents the linear change rate of absorbance during the reaction, T represents the reaction time (min), Vt represents the total volume of crude enzyme solution (mL), Vs represents the volume of enzyme solution used for measurement (mL), and FW represents the fresh weight of the sample (g).

The malondialdehyde (MDA) content was determined according to the method of Oltra et al. [30] with appropriate modifications. Fresh cucumber leaves (0.5 g) were homogenized in 5 mL of ice-cold 10% trichloroacetic acid (TCA) and centrifuged at 10,000× *g* at 4 °C for 10 min. Take 2 mL of the supernatant and mix it with an equal volume of 0.5% thiobarbituric acid (TBA) solution in a test tube. Place the mixture in a boiling water bath for 30 min, then immediately transferred it to ice to terminate the reaction and cool it. Measure the absorbance values at wavelengths of 532 nm and 600 nm. The MDA content was calculated as follows: MDA content (μmol·g^−1^) = [(ΔA_532_ − ΔA_600_) × V × A]/(a × 0.155 × FW), where ΔA_532_ and ΔA_600_ represent the absorbance values at 532 nm and 600 nm, respectively; A is the total volume of the reaction mixture (mL); a is the volume of extract used in the assay (mL); V is the total volume of the TCA extract (mL); and FW is the fresh weight of the sample (g).

The activity of ascorbate peroxidase (APX) was determined following the method of Chen et al. [31] with appropriate modifications. One milliliter of the supernatant obtained above was used as the enzyme extract. To this, 1 mL of 0.1 mM H_2_O_2_ solution, 0.1 mL of enzyme extract, 1.8 mL of 50 mM phosphate buffer, and 0.1 mL of 150 mM ascorbic acid (AsA) solution were added. After standing at room temperature for 3 min, the mixture was thoroughly mixed, and the change in absorbance (A_290_) at 290 nm was recorded within 10 to 30 s. The APX activity was calculated based on the rate of ascorbic acid oxidation. The formula for total APX activity (U·g^−1^·min^−1^) is: APX total activity = △OD × Vr/(A × d × Vt × W × △t), where △OD is the change in absorbance during the reaction time, △t is the reaction time in minutes, Vr is the volume of the extract in mL, A is the extinction coefficient, 2.8 mM^−1^·cm^−1^, d is the path length of the cuvette, 1 cm, Vt is the volume of the reaction mixture in mL, and W is the fresh weight of the sample in g.

The glutathione (GSH) content was determined as follows: approximately 0.1 g of fresh cucumber leaves was accurately weighed, ground into a fine powder under liquid nitrogen, and extracted according to the manufacturer’s instructions for the Solarbio Glutathione Assay Kit (Product Code: BC1175, Solarbio Science & Technology Co., Ltd., Beijing, China) for quantitative analysis of GSH content. Cucumber root and leaf tissues were rinsed twice with phosphate-buffered saline (PBS), followed by accurate weighing of 0.1 g plant sample. The tissue was transferred to a pre-chilled glass homogenizer that had been rinsed with Reagent One, and 1 mL of Reagent One was added (maintaining a constant tissue-to-reagent volume ratio). Homogenization was performed rapidly on ice (with improved cell disruption efficiency when using liquid nitrogen). The homogenate was centrifuged at 8000× *g* and 4 °C for 10 min, and the supernatant was collected and stored at 4 °C until analysis. For the assay, 100 μL of diluted standard or test sample was added to a 1.5 mL centrifuge tube, followed by 2 μL of Reagent Two. The mixture was vortexed thoroughly and incubated at 37 °C for 30 min. Subsequently, 700 μL of Reagent Three, 100 μL of Reagent Four, 100 μL of Reagent Five, and 10 μL of Reagent Six were added sequentially to the reaction tube (timing initiated immediately upon addition of Reagent Six). After rapid mixing, absorbance was measured at 412 nm at 30 s and 150 s after reagent addition (recorded as A_1_ and A_2_, respectively), and the change in absorbance (ΔA) was calculated as ΔA = A_2_ − A_1_.

### 2.7. Determination of Cadmium Content

Root systems and leaves of cucumber seedlings were collected. The samples were initially washed three times with distilled water. Subsequently, the root tissues were immersed in an EDTA solution (prepared by dissolving 29.2240 g of EDTA powder in 1 L of distilled water) for 15 min. Following this treatment, all samples were rinsed an additional three times with distilled water, surface moisture was removed using filter paper, and the tissues were transferred to small beakers. These beakers were placed in a drying oven maintained at 80 °C for a duration of 36 h. After drying the tissue samples, the roots and leaves were ground into fine powder and packaged in labeled envelopes for sealed storage for subsequent experimental analysis. For further processing, 0.5000 g (accurate to four decimal places) of each powdered sample was precisely weighed, transferred into a 100 mL beaker, covered with a 60 mm watch glass, and appropriately labeled. The samples were digested using a mixed acid system composed of concentrated HNO_3_ and 30% H_2_O_2_ in a volume ratio of 4:1. The samples were immersed in beakers for 24 h and then heated on an electric hot plate at 200 °C for about 5 h. During the digestion process, the same proportion of digestion acid solution (concentrated HNO_3_ and 30% H_2_O_2_, volume ratio 4:1) was replenished in a timely manner, and the reaction temperature was controlled to avoid violent reactions or solution splashing. At the same time, acid evaporation was carried out (when a large amount of white smoke was produced in the beaker, the surface dish could be slightly opened to promote acid evaporation) until the solution was clear and transparent with no obvious residue. After digestion, the solution was made up to 20 mL with 1% HNO_3_ and transferred to a 50 mL centrifuge tube, and stored in a refrigerator at 4 °C for further determination [32]. Cd concentrations in both plant tissues and soil were measured using an atomic absorption spectrophotometer (PerkinElmer, Waltham, MA, USA).

The translocation factor (TF) indexes of cucumber were further calculated as follows:

TF = Cd concentration in shoot/Cd concentration in root.

TF = Cd concentration in shoot/Cd concentration in leave.

### 2.8. Quantitative Real-Time RT-qPCR

Plant-specific RNA extraction kit (TianGen Bio RNAprep Pure Plant, Beijing, China) was used. Samples were ground in liquid nitrogen and homogenized with lysis buffer. RNA was purified using a spin column and treated with DNase to remove genomic DNA contamination. Reverse transcription was performed using a gDNase-containing reverse transcription kit (TianGen FastQuant RT Kit, model KR106, Beijing, China) with Oligo (dT) or random primers at 42 °C for 15 min, followed by inactivation at 95 °C. Real-time quantitative PCR (qPCR) amplification and fluorescence detection were carried out using SuperReal PreMix Plus (TianGen SYBR Green, model FP205, Beijing, China). The reaction system (10 μL) included 4.5 μL of 2 × SYBR Green premix, 0.1 μL of forward and reverse primers each, and 1 μL of cDNA template. Primers were designed using the QuantPrime tool (http://quantprime.mpimp-golm.mpg.de/, accessed on 14 March 2025). The amplification program was 95 °C for 15 min for pre-denaturation → 40 cycles (95 °C for 10 s → 60 °C for 20 s → 72 °C for 1 min) → melting curve analysis (95 °C → 60 °C → 72 °C gradient detection). Cucumber actin gene was used as the internal reference gene [33]. The sequences of the primers used are detailed in Supplementary Table S1.

### 2.9. Data Processing and Analysis

Data analysis was performed using Excel 2021 and SPSS 21.0 software, while Origin 2024 was employed for graph generation. Significant differences among treatments were analyzed using Duncan’s multiple range test, with statistical significance set at *p* < 0.05. Different lowercase letters denote statistically significant differences between treatment groups.

## 3. Results

### 3.1. Regulatory Effect of Eexogenous Spd Spraying on the Inhibition of Cucumber Seeds Germination and Growth Under Cd Stress

**Figure 1:**
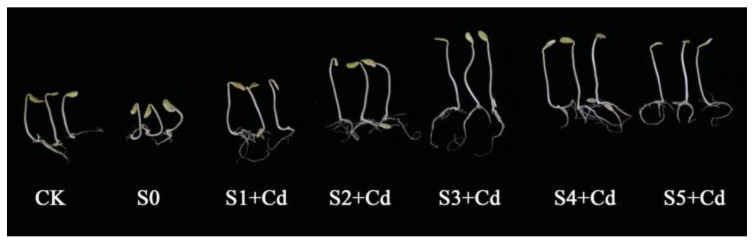
The effect of exogenous Spd on growth indices of cucumber seeds under Cd stress. CK: Control group (distilled water); S0: 10 mg·L^−1^ Cd treatment; S1+ Cd: 10 mg·L^−1^ Cd + 0.05 mM Spd group; S2+ Cd: 10 mg·L^−1^ Cd + 0.1 mM Spd group; S3+ Cd: 10 mg·L^−1^ Cd + 0.2 mM Spd group; S4+ Cd: 10 mg·L^−1^ Cd + 0.4 mM Spd group; S5+ Cd: 10 mg·L^−1^ Cd + 0.5 mM Spd group.

Phenotypic observations of cucumber seeds treated with varying concentrations of Spd for 7 days revealed that, as illustrated in Figure 1, compared to the control group (CK) and the cadmium (Cd)-stressed group without Spd (S0), Spd treatments at concentrations of 0.05 mM, 0.1 mM, 0.2 mM, 0.4 mM, and 0.5 mM exerted differential growth-promoting effects under Cd stress. As shown in Table 1, the S0 group, subjected to 10 mg·L^−1^ Cd without Spd, exhibited significantly reduced germination potential and germination rate compared to the CK group (0 mg·L^−1^ Cd + 0 mM Spd), indicating that Cd exerts inhibitory effects on cucumber seed germination. When Spd was applied at 0.2 mM (S3 + Cd), it significantly alleviated Cd-induced stress, increasing germination potential and rate by 16.67% and 15.56%, respectively, compared to S0. This suggests that an appropriate concentration of Spd can mitigate Cd toxicity and enhance germination performance. However, at Spd concentrations of 0.4 mM and 0.5 mM, both germination parameters were significantly lower than those of CK, showing decreases of 3.33% and 1.11%, and 2.22% and 2.22%, respectively, indicating that high Spd concentrations inhibit germination. At 0.1 mM (S2 + Cd), Spd significantly enhanced germination potential and rate compared to CK, demonstrating the most pronounced promoting effect. These findings consistent with the results reported by Liu et al. [34]. Further observations revealed that exogenous Spd application under Cd stress improved germination parameters to varying degrees. Notably, at 0.2 mM (S3 + Cd), germination potential and rate increased by 24.48% and 19.50%, respectively, compared to S0.

Root length and sprout length measurements on day 7 are presented in Table 1. Under Cd stress (S0) and Cd + Spd treatments, both parameters exhibited a trend of initial increase followed by a decline with increasing Spd concentration. In the S0 group, root length was 5.08 cm, which increased to 8.24 cm under 0.2 mM Spd (S3 + Cd), representing a 62.27% increase. Similarly, sprout length increased from 5.02 cm in S0 to 7.21 cm under 0.2 mM Spd, a 43.62% improvement. At 0.5 mM Spd, sprout length increased by 26.69% compared to S0. These results indicate that moderate Spd concentrations enhance root and sprout development under Cd stress, while excessive concentrations diminish the promoting effect. In the absence of Cd stress, exogenous Spd at 0.1 mM (S2) significantly increased root and sprout lengths by 12.85% and 8.01%, respectively, compared to CK, further supporting the growth-promoting role of optimal Spd levels.

Data from Table 1 also indicate that under Cd stress alone (S0), the fresh and dry weights of cucumber seedlings were 1.7565 g and 0.1805 g, respectively. With increasing Spd concentration, both parameters improved, peaking at 0.1 mM Spd (S2 + Cd), where fresh weight reached 2.0405 g and dry weight reached 0.2017 g—representing increases of 16.17% and 11.75% compared to S0. These differences were statistically significant. When Spd concentration was increased to 0.5 mM, fresh and dry weights slightly decreased to 2.0008 g and 0.1914 g, respectively. These findings suggest that Spd can enhance biomass accumulation under Cd stress within a certain concentration range, but its efficacy diminishes at higher concentrations.

### 3.2. Effects of Varying Spd Concentrations on Phenotypic Traits and Osmotic Regulatory Substances in Cucumber Seedlings Under Cd Stress

**Figure 2:**
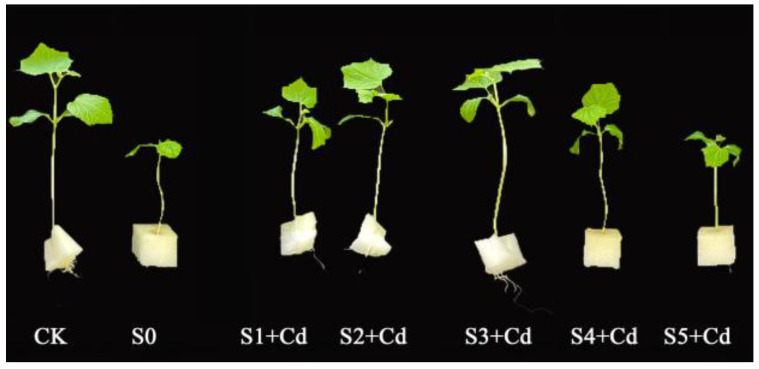
The effect of exogenous Spd on the phenotype of cucumber seedlings under cadmium stress. CK: Control group (distilled water); S0: 10 mg·L^−1^ Cd treatment; S1+ Cd: 10 mg·L^−1^ Cd + 0.05 mM Spd group; S2+ Cd: 10 mg·L^−1^ Cd + 0.1 mM Spd group; S3+ Cd: 10 mg·L^−1^ Cd + 0.2 mM Spd group; S4+ Cd: 10 mg·L^−1^ Cd + 0.4 mM Spd group; S5+ Cd: 10 mg·L^−1^ Cd + 0.5 mM Spd group.

Phenotypic observations of treated cucumber seedlings are presented in Figure 2. Exogenous Spd was found to alleviate the inhibitory effects of Cd stress on seedling growth, with the most pronounced ameliorative effect observed at moderate Spd concentrations. Notably, the S3 + Cd treatment group exhibited improved seedling morphology, with stem and leaf development closely resembling those of the control group (CK). The protective effect of exogenous Spd against Cd stress increased with rising Spd concentration up to a certain threshold. However, excessively high Spd concentrations may induce adverse physiological responses, thereby negatively affecting seedling growth.

**Figure 3:**
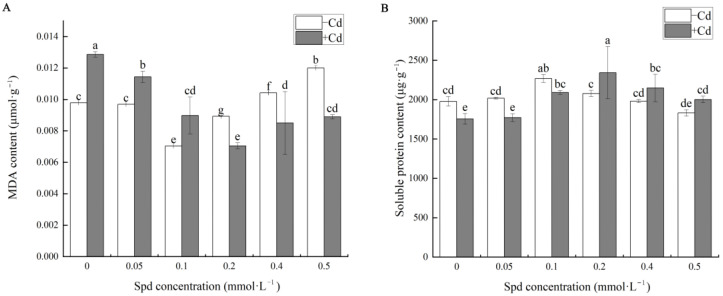
Effects of exogenous Spd on the content of Osmotic adjustment substances in leaves of cucumber seedlings under Cd stress. (**A**) Shows the contents of Malondialdehyde (MDA). (**B**) Shows the contents of soluble protein. Note: Values are means ± SD, *n* = 3; different letters indicate significant differences (*p* < 0.05).

The levels of osmotic regulatory substances were subsequently analyzed in Figure 3A. Under CK conditions, the malondialdehyde (MDA) content in cucumber seedlings remained at a relatively low baseline. Compared to the non-Cd control, Cd exposure in the absence of Spd (S0 group) significantly elevated MDA levels by approximately 31.46%, indicating increased oxidative damage under Cd stress. In the presence of exogenous Spd, MDA content decreased significantly. The lowest MDA level was observed at an Spd concentration of 0.2 mM (S3 + Cd), representing a marked reduction compared to Cd stress alone. When Spd concentration reached 0.5 mmol·L^−1^, MDA content remained lower than that under Cd stress alone but was significantly higher than at 0.2 mmol·L^−1^, showing a 27.14% increase. These findings suggest that appropriate Spd concentrations effectively mitigate Cd-induced oxidative damage by reducing lipid peroxidation, with 0.2 mM Spd yielding the most favorable outcome. However, excessive Spd may diminish its protective efficacy.

As illustrated in Figure 3B, Cd stress significantly altered the soluble protein content in cucumber seedlings. Compared to the CK group, Cd-treated seedlings exhibited notable fluctuations in soluble protein levels with increasing Spd concentration. The highest soluble protein content was observed at 0.2 mM Spd, which was 33.43% higher than that in the Cd-only group (S0), indicating enhanced stress tolerance through increased protein synthesis. At 0.5 mM Spd, soluble protein content remained significantly higher than S0 (by 14.05%), although it was lower than at 0.2 mM. These results demonstrate that Spd can modulate soluble protein accumulation under Cd stress, thereby enhancing the seedlings’ capacity for cellular self-regulation and stress mitigation. However, when Spd concentration exceeds the optimal range, its regulatory capacity may decline, potentially compromising the seedlings’ ability to maintain cellular homeostasis.

### 3.3. Effects of Varying Spermidine Concentrations on Superoxide Dismutase (SOD), Peroxidase (POD), Catalase (CAT), and Ascorbate Peroxidase (APX) Activities in Cucumber Seedlings Under Cd Stress

**Figure 4:**
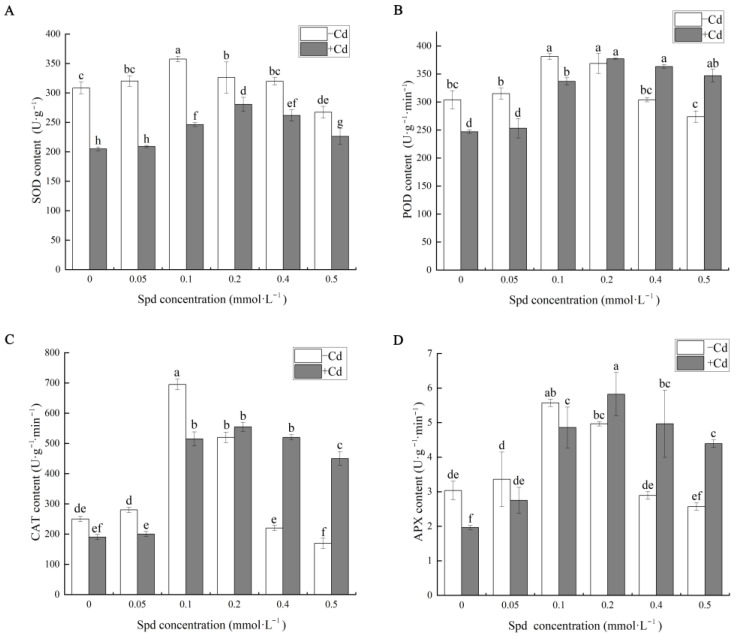
The exogenous Spd application alleviates salt stress by scavenging ROS accumulation in leaves of cucumber seedlings under Cd stress. (**A**) Shows the contents of superoxide (SOD). (**B**) Shows the contents of peroxidase (POD). (**C**) Shows the contents of catalase (CAT). (**D**) Shows the contents of Ascorbate peroxidase (APX). Note: Values are means ± SD (*n* = 3); different letters indicate significant differences (*p* < 0.05).

Plants can mitigate cadmium (Cd)-induced oxidative damage by enhancing the activities of key antioxidant enzymes, including superoxide dismutase (SOD), peroxidase (POD), catalase (CAT), and ascorbate peroxidase (APX). Under adverse environmental stress, excessive reactive oxygen species (ROS) accumulate in plant cells, leading to oxidative stress. These antioxidant enzymes play central roles in scavenging of reactive oxygen species and are critical components of the plant’s defense mechanisms against abiotic stress [35]. As illustrated in Figure 4, exogenous application of spermidine (Spd) effectively modulates the activities of these antioxidant enzymes in cucumber seedlings under Cd stress.

Compared with the control group (CK), Cd exposure obviously altered the enzymatic contents of SOD, POD, CAT, and APX. Specifically, under 10 mg·L^−1^ Cd stress, the activities of SOD, POD, CAT, and APX decreased by 22.16%, 50.49%, 5.26%, and 54.54%, respectively, relative to CK. This decline may be attributed to the displacement of essential metal ions in the active centers of these enzymes by Cd^2+^, thereby impairing their catalytic functions. When Spd was applied in the absence of Cd stress, the activities of these enzymes exhibited distinct concentration-dependent trends. For example, at a low Spd concentration of 0.1 mM, certain enzyme activities were significantly enhanced. However, at a high Spd concentration of 0.5 mM, enzyme activities were partially suppressed. These findings indicate that an appropriate Spd concentration can significantly enhance antioxidant enzyme activities, whereas excessive Spd may exert inhibitory effects.

In the Cd-stressed groups supplemented with Spd, the activities of SOD, POD, CAT, and APX varied in a concentration-dependent manner, showing an initial increase followed by a decrease with increasing Spd levels. Among all treatments, 0.2 mM Spd (S3 + Cd) demonstrated the most pronounced alleviating effect. Compared to Cd stress alone, the addition of 0.2 mM Spd significantly increased the activities of SOD, POD, CAT, and APX by 29.08%, 36.83%, 192.11%, and 196.36%, respectively. These results suggest that optimal Spd application can markedly enhance antioxidant defense systems, thereby mitigating Cd-induced oxidative damage in cucumber seedlings. However, inappropriate Spd concentrations may suppress enzymatic activity, disrupt normal physiological processes, and ultimately hinder seedling growth and development under Cd stress conditions.

### 3.4. Effects of Different Concentrations of Spd on Cadmium Accumulation Indicators in Cucumber Seedlings Under Cd Stress

When the Cd concentration was 10 mg·L^−1^, the cadmium content in the roots was significantly higher than that in the aboveground parts, indicating that the roots were the main accumulation site of cadmium (Table 2). The cadmium content in the roots of all treatment groups (S0 + Cd-S5 + Cd) ranged from 597.5 to 1261.1 mg/kg. With the increase in Spd concentration (0–0.2 mM), the cadmium content in the roots gradually decreased (S0-S3), reaching the lowest at 0.2 mM Spd (S3 + Cd). However, when the Spd concentration continued to increase (0.4–0.5 mM), the cadmium content rebounded. The possible reason is that low concentrations of Spd alleviate cadmium toxicity, but high concentrations may induce physiological stress, leading to a recovery in cadmium absorption. The trend of cadmium content change in the leaves was consistent with that in the roots, but the absolute values were lower (227.71–88.45 mg/kg).

The cadmium content was the lowest at 0.2 mM Spd (S3 + Cd) (88.45 mg/kg), which was 61.16% lower than that of S0 (without Spd). Within the range of 0.05–0.2 mM Spd, the cadmium accumulation decreased with the increase in Spd concentration; however, it slightly rebounded when the Spd concentration exceeded 0.2 mM (S4 + Cd-S5 + Cd). The translocation factor (aboveground part/root) reflects the transport ability of cadmium from the roots to the aboveground parts. All treatment groups (0.148–0.18) were significantly lower than S0 (0.18), but only S3 + Cd (0.148) and S4 + Cd-S5 + Cd (0.151) showed significant differences from S0. This indicates that Spd may inhibit the transport of Cd from the roots to the aboveground parts, especially at 0.2 mM, the effect was most obvious.

### 3.5. Correlation Analysis of Exogenous Spd Treatment on Cd-Stressed Cucumber Seedlings

**Figure 5:**
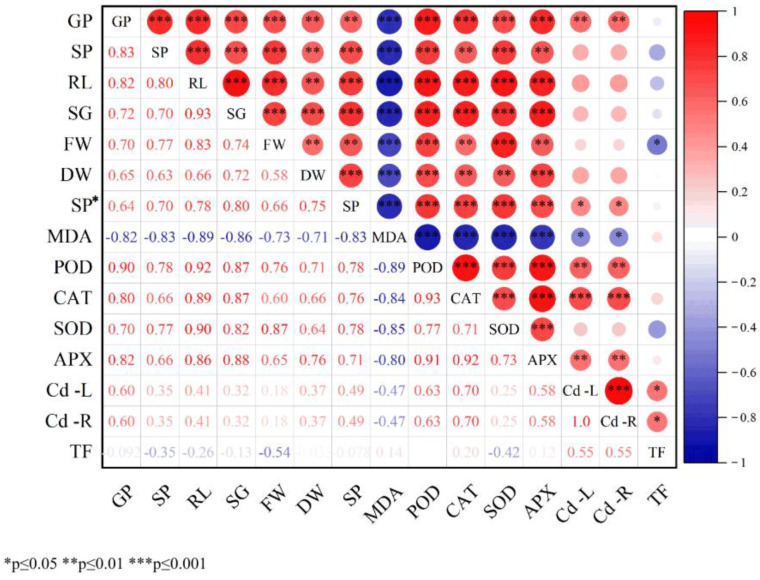
Correlation analysis of changes in various indicators of cucumber seedlings induced by exogenous Spd under Cd Stress. Note: GR, germination rate; GP, germination potential; RL, root length; SG, length of the young sprout; FW, fresh weight; DW, dry weight; SP*, soluble protein; MDA, malondialdehyde; POD, peroxidase; CAT, catalase; SOD, superoxide dismutase; APX, ascorbate peroxidase; Cd-L, the cadmium content in the leaves, Cd-R, the cadmium content in the roots, and TF for the transfer coefficient. * *p* ≤ 0.05, ** *p* ≤ 0.01, *** *p* ≤ 0.001. Red tones indicate positive correlations (positive correlation coefficients ranging from light red to dark red, corresponding to correlation coefficients from 0 to 1, with darker shades representing stronger positive correlations). Blue tones indicate negative correlations (negative correlation coefficients ranging from light blue to dark blue, corresponding to correlation coefficients from 0 to −1, with darker shades representing stronger negative correlations). White represents the absence of a statistically significant correlation (correlation coefficient close to 0 or non-significant correlation).

Correlation analysis among all measured parameters is presented in Figure 5, revealing predominantly highly significant positive correlations. The correlation coefficient between germination percentage (GP) and peroxidase (POD) activity was notably high (0.90), suggesting a strong positive feedback relationship between germination capacity and antioxidant defense mechanisms. Similarly, root length (RL) showed strong positive correlations with the length of the young sprout (SG, 0.93), POD (0.92), and superoxide dismutase (SOD, 0.90), indicating that root development is closely associated with germination potential and antioxidant enzyme activities. The correlation between POD and catalase (CAT) was 0.93, and between CAT and ascorbate peroxidase (APX) was 0.92, demonstrating a high degree of synergy among antioxidant enzymes in scavenging reactive oxygen species under stress conditions. With the exception of total malondialdehyde (MDA) and transfer factor (TF), all other indices exhibited positive correlations. MDA, however, showed significant negative correlations with several parameters, including GP, Sprouting potential (SP), RL, SG, fresh weight (FW), dry weight (DW), soluble protein (SP*), RL, SG, POD, CAT, SOD, APX, Cd content in leaves (Cd-L), and Cd content in roots (Cd-R). Two particularly strong negative correlations were observed: between MDA and RL (−0.89), indicating oxidative damage to root elongation, and between MDA and POD (−0.89), reflecting impaired antioxidant enzyme accumulation. Overall, the majority of the measured indices displayed consistent variation trends, with evident interrelationships and overlapping effects. These findings suggest the presence of complex interactions among physiological, biochemical, and growth-related indicators. Therefore, multivariate statistical approaches are recommended for further comprehensive analysis of the entire dataset to better understand the underlying mechanisms.

### 3.6. Cluster Analysis of Cd-Stressed Cucumber Seedlings Treated with Exogenous Spd

**Figure 6:**
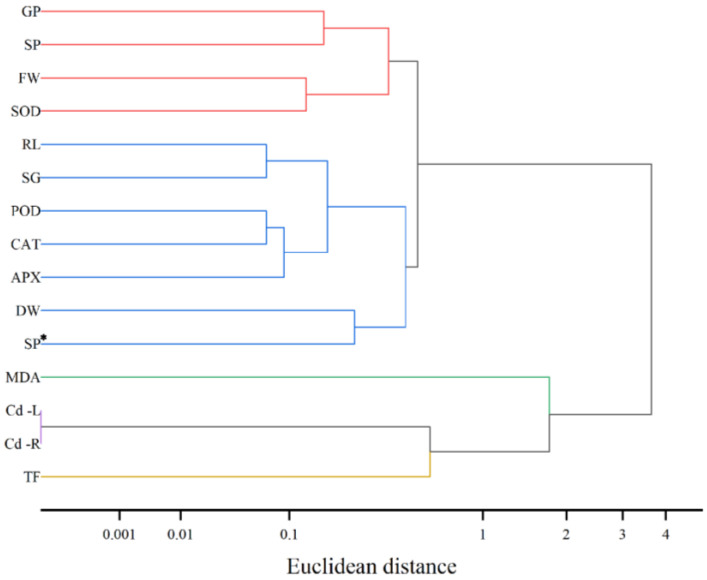
Cluster analysis of matrix in various indicators of cucumber seedlings induced by exogenous Spd under Cd Stress. Note: GR, germination rate; GP, germination potential; RL, root length; SG, length of the young sprout; FW, fresh weight; DW, dry weight; SP*, soluble protein; MDA, malondialdehyde; POD, peroxidase; CAT, catalase; SOD, superoxide dismutase; APX for ascorbate peroxidase; Cd-L for the cadmium content in the leaves, Cd-R for the cadmium content in the roots, and TF for the transfer factor. Clustering results are differentiated by colors (red, blue, green, purple, and orange), where identical colors indicate membership in the same cluster in the figure. Variables within a cluster are grouped based on highly similar data features, as evidenced by shorter Euclidean distances and higher Pearson correlation coefficients.

The research findings demonstrate (Figure 6) that systematic clustering categorized the 15 measured indicators into five distinct clusters. The first cluster, representing germination-related parameters (GP, SP, FW, and SOD), exhibited tight clustering (distance < 0.1), indicating a highly synergistic response of germination rate, germination potential, fresh weight, and superoxide dismutase activity to cadmium stress. The second cluster encompassed both growth-related and antioxidant-related indicators (RL, SG, POD, CAT, APX, DW, and SP*), which were grouped together, suggesting their interrelated roles in antioxidant processes and their combined influence on the growth of cucumber seedlings. The third cluster included cadmium content-related indicators (MDA, Cd-L, and Cd-R), reflecting the correlation between malondialdehyde levels, leaf cadmium content, and root cadmium content, and indicating cadmium accumulation and distribution within the seedlings. The fourth cluster consisted solely of the transfer factor (TF), highlighting its independence and unique role in the response to cadmium stress. Overall, the hierarchical response pattern of exogenous spermidine under cadmium stress in cucumber seedlings reveals that various physiological and biochemical indicators respond in a coordinated or independent manner across different levels, collectively forming a complex regulatory network.

### 3.7. Principal Component Analysis of Exogenous Spd Treatment on Cd Stressed Cucumber Seedlings

The principal component analysis method was used to comprehensively analyze the following 15 indicators to evaluate the overall impact of different indicators on cucumber seedlings. As shown in Table 3, the maximum eigenvalue was 10.16796, the maximum contribution rate was 67.79%, and the cumulative contribution rate of the first three principal components was 89.49%, exceeding 85.000%. Therefore, the first three components could be extracted as the indicators for the subsequent membership function analysis.

**Figure 7:**
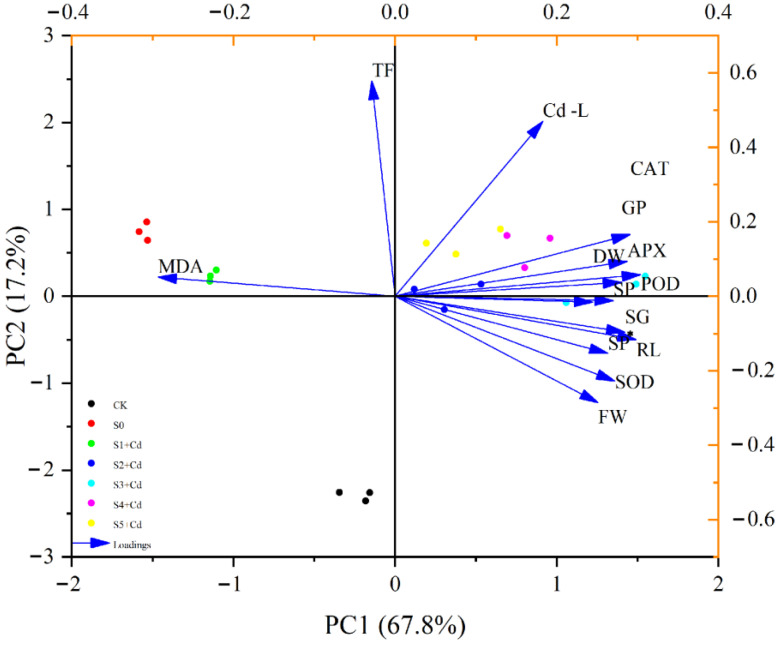
The principal component analysis (PCA) loading plot illustrates the distribution of samples within the two-dimensional factor space defined by PC1 and PC2. Distinct colors correspond to different experimental groups, with observed clustering patterns highlighting significant inter-group variations. The black dots (CK) denote the control group, serving as a reference baseline for evaluating treatment-induced effects. Red (S0), Green (S1 + Cd), Blue (S2 + Cd), Cyan (S3 + Cd), Purple (S4 + Cd), and Yellow (S5 + Cd). Arrows represent different variables or biochemical indicators. The direction of each arrow indicates the sign of the correlation between a given variable and the principal components, while the length reflects its relative contribution to the component structure. Colored dotsdenote distinct samples or treatment groups. Color coding differentiates experimental conditions or categories, and the spatial positions of the points illustrate their distribution within the principal component space.

PCA biplot analysis of principal component analysis: As shown in Figure 7, principal component analysis revealed that PC1 and PC2 accounted for 67.8% and 17.2% of the total variance, respectively, with a cumulative explained variance of 84.95%, indicating that these two components effectively capture the majority of the variation within the dataset. The S0 (Cd stress) and S1 + Cd (0.05 mM Spd + Cd) were predominantly clustered along the negative axis of PC1, with their distribution closely aligned with the direction of the malondialdehyde (MDA) vector, indicating that seedlings subjected to these treatments experienced severe oxidative damage. S2 + Cd (0.1 mM Spd + Cd), S3 + Cd (0.2 mM Spd + Cd), S4 + Cd (0.4 mM Spd + Cd), and S5 + Cd (0.5 mM Spd + Cd) were all distributed along the positive axis of PC1 (in the same direction as the FW/SOD/RL arrows). From Figure 7, it can be seen that the distribution of the treatment groups S1-Cd to S5-Cd on PC1 was relatively dispersed, indicating that these treatments had different effects on the variables. The CK group was clustered near the origin, indicating a relatively stable physiological homeostasis under non-stressed conditions.

### 3.8. Comprehensive Evaluation by Membership Function

The PCA (Table 4) showed that the D value was the weighted average of the membership degrees, which integrated the membership degrees of the three principal components to generate an overall evaluation index (range 0–1), with a higher value indicating better overall performance of the treatment group. The weights w1 = 0.757, w2 = 0.192, and w3 = 0.051 were the variance contribution rates of the principal components, indicating that PAC1 had the strongest explanatory power for the overall variation (75.7%), followed by PAC2 (19.2%), and PAC3 had the smallest contribution (5.1%). Based on the weights, PAC1 (with a high contribution rate) might be associated with core stress response indicators (such as biomass or enzyme activity), and its membership degree (X1) increased with the increase in Spd concentration; the membership degrees of PAC2 and PAC3 changed slightly and had a weaker contribution, reflecting secondary physiological processes. S3 + Cd (0.2 mM Spd) had the highest score (0.939), indicating that this concentration of Spd had the best effect in alleviating cadmium stress. Through comprehensive evaluation, it was concluded that: S3 + Cd > S4 + Cd > S2 + Cd > S5 + Cd > CK > S1 + Cd > S0, and 0.2 mM Spd was recommended as the optimal concentration.

### 3.9. Detection and Analysis of the Expression of Related Genes in Cucumber Seedlings Under Cd Stress Treated with Exogenous Spd

**Figure 8:**
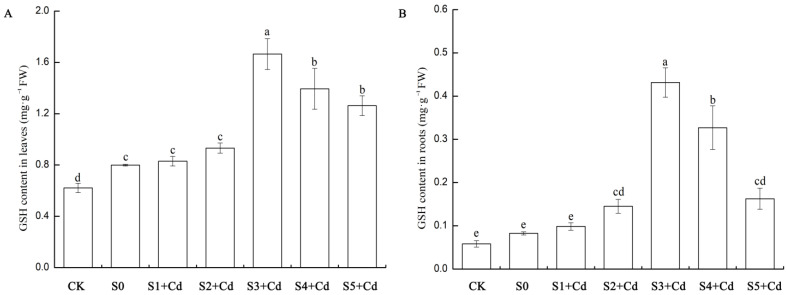
Effects of exogenous Spd on GSH content in cucumber seedlings under Cd stress. (**A**) leaves; (**B**) roots. different letters indicate significant differences (*p* < 0.05).

When plants are exposed to cadmium stress, they activate multiple defense mechanisms to resist toxic damage, and these mechanisms are regulated at the molecular level by related genes. In this study, the content of glutathione (GSH) in the roots and leaves of cucumber seedlings treated with different concentrations of Spd after cadmium stress was determined. The results are shown in Figure 8. In cucumber leaves, compared with CK, the GSH content increased by 29% after cadmium stress, indicating that cadmium stress systemically induces antioxidant responses. After exogenous application of Spd, the GSH content in cucumber leaves increased. Compared with the S0 treatment group, the GSH content slightly increased in the S1 + Cd and S2 + Cd groups at low concentrations of Spd (0.05–0.1 mM), but there was no significant difference. At a medium concentration of 0.2 mM Spd (S3 + Cd), the GSH content increased by 107% compared with S0 and was significantly higher than other groups. At high concentrations of Spd (0.4–0.5 mM), the GSH content gradually decreased but was still significantly higher than S0 (Figure 8A). In the roots, compared with the control CK, the GSH content increased by 33% after cadmium treatment. After exogenous application of Spd, the GSH content in the roots also increased. At low concentrations of Spd (0.05–0.1 mM), the GSH content slightly increased in the S1 + Cd and S2 + Cd groups, but there was no significant difference. At a medium concentration of 0.2 mM Spd (S3 + Cd), the GSH content increased to 0.43 (438% higher than S0), which was significantly higher than other groups. At high concentrations of Spd (0.4–0.5 mM), the GSH content decreased but was still significantly higher than S0 (Figure 8B), indicating that the promotion of GSH in the roots by Spd is strongly concentration-dependent, with 0.2 mM being the optimal concentration.

**Figure 9:**
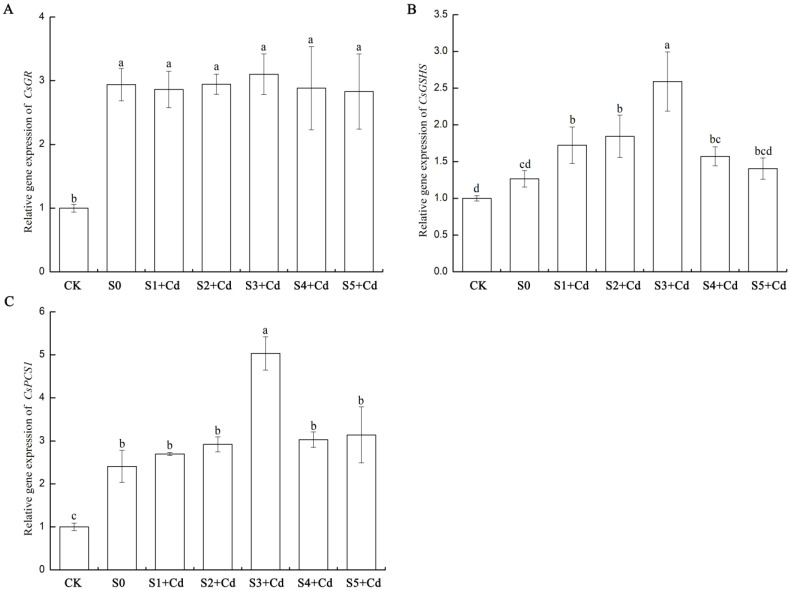
Effects of exogenous Spd on the expression of genes related to GSH pathway in cucumber seedlings under Cd stress. (**A**) show the relative expression of *CsGR* gene. (**B**) show the relative expression of *CsGSHS* gene. (**C**) show the relative expression of *CsPCS1* gene. Note: Data were expressed as the mean ± standard deviation (SD) from three independent biological replicates. Different lowercase letters denoted statistically significant differences among treatment groups at *p* < 0.05. CK: Distilled water as a control, S0: 10 mg·L^−1^ Cd treatment; S1 + Cd: 10 mg·L^−1^ Cd + 0.05 mM Spd group; S2+ Cd: 10 mg·L^−1^ Cd + 0.1 mM Spd group; S3+ Cd: 10 mg·L^−1^ Cd + 0.2 mM Spd group; S4+ Cd: 10 mg·L^−1^ Cd + 0.4 mM Spd group; S5+ Cd: 10 mg·L^−1^ Cd + 0.5 mM Spd group.

As shown in Figure 9, compared with CK, the expression of *CsGR* (XM_011652579.2) was upregulated in all treatments. The expression of *CsGR* in the S0 (only cadmium stress) group increased by 193.9%, indicating that cadmium stress significantly activates the antioxidant system. CsGR maintains the intracellular GSH/GSSG balance by reducing oxidized glutathione (GSSG) to reduced GSH. At low concentrations of Spd, the expression of CsGR in the S1 + Cd treatment group slightly decreased, possibly because Spd partially replaced the antioxidant function of GSH. The expression of *CsGR* in the S2 + Cd treatment group was the same as that in S0, indicating that low concentrations of Spd did not significantly interfere with CsGR activity. The expression of *CsGR* in the S3 + Cd treatment group at a medium concentration of Spd reached its peak, although it was not significantly higher than S0, the value was the highest, possibly due to the synergistic enhancement of antioxidant capacity with the peak GSH content. Compared with S0, the expression of *CsGR* increased in the high concentration Spd (S4 + Cd-S5 + Cd) treatment groups, but slightly decreased, still significantly higher than CK, possibly because the antioxidant effect of high concentration Spd itself reduced the dependence on CsGR. In summary, the study found that cadmium stress significantly induced the expression of *CsGR*, but exogenous Spd (0.05–0.5 mM) did not further significantly increase its expression level.

Compared with CK, the expression of *CsGSHS* in cucumber leaves under S0 (only cadmium stress) increased by 26.6%, indicating that cadmium stress slightly activated the expression of the glutathione synthetase (GSHS) gene, but did not reach a significant level. At low concentrations of Spd (0.05–0.1 mM), the expression of *CsGSHS* (HM230748.1) in the S1 + Cd treatment group increased by 36% compared with S0, and further increased by 46% in the S2 + Cd treatment group, indicating that low concentrations of Spd significantly promoted the expression of *CsGSHS*; at a medium concentration of Spd (0.2 mM, S3 + Cd), the expression of *CsGSHS* increased by 105% compared with S0 and was significantly higher than other groups; When the concentration of Spd was high (0.4–0.5 mM), the expression level of *CsGSHS* slightly decreased. However, the expression level in the S4 + Cd treatment group was still significantly higher than that in the S0 group, while there was no significant difference between the S5 + Cd treatment group and the S0 group. This indicates that there is an upper limit to the promoting effect of Spd, and the effect weakens when the concentration exceeds 0.2 mM.

Compared with CK, the expression level of *CsPCS1* (XM_004140521.3) in the cucumber leaves of the S0 group (only Cd stress) increased by 141%, indicating that Cd stress significantly activates the expression of the plant chelate synthase 1 (*PCS1*) gene, which is a typical response of plants to heavy metal toxicity (PCS1 catalyzes the synthesis of phytochelatins (PCs) for chelating Cd). When the concentration of Spd was low (0.05–0.1 mM), the expression level of *CsPCS1* in the S1 + Cd treatment group increased by 12% compared with the S0 group, and in the S2 + Cd treatment group, it further increased by 21%, indicating that the promoting effect of low concentration Spd on *CsPCS1* was relatively weak. When the concentration of Spd was medium (0.2 mM, S3), the expression level of CsPCS1 increased by 109% compared with the S0 group, which completely corresponded to the peak value of GSH content (GSH in S3 + Cd leaves = 1.66) and the lowest value of Cd accumulation (Cd in the aboveground part of S3 + Cd = 88.45 mg/kg), indicating that Spd enhances Cd chelation and compartmentalization by synergistically up-regulating *PCS1* and GSH synthesis. When the concentration of Spd was high (0.4–0.5 mM), the expression level of *CsPCS1* in the S4 + Cd treatment group slightly decreased but was still higher than that in the S0 group, while in the S5 + Cd treatment group, the expression level of *CsPCS1* slightly increased, possibly due to the slight stress caused by the excessively high concentration of Spd on the plants.

Data were expressed as the mean ± standard deviation (SD) from three independent biological replicates. Different lowercase letters denoted statistically significant differences among treatment groups at *p* < 0.05.

## 4. Discussion

Cd is a non-essential and highly toxic heavy metal that poses significant risks to ecological systems and human health once it enters the food chain. As foundational components of the food chain, plants play a critical role in maintaining ecosystem stability and environmental safety. Upon Cd uptake, plants experience a range of physiological and biochemical disturbances, with biomass alteration being one of the most direct and observable effects. Preliminary experimental results demonstrated that when Cd concentrations were set at 0, 0.25, 0.5, 1.0, 3.0, 5.0, and 10.0 mg·L^−1^, the impact on cucumber seed biomass aligned with previous findings—low Cd concentrations exhibited stimulatory effects, while high concentrations were inhibitory. Notably, a Cd concentration of 10.0 mg·L^−1^ significantly suppressed seed growth. Therefore, this concentration was selected for subsequent experimental evaluations of Cd-induced stress in cucumber seeds and seedlings. The application of exogenous spermidine (Spd) partially alleviated the adverse effects of Cd stress on biomass (Figure 1, Table 1). However, this ameliorative effect was statistically significant only under treatment with 0.2 mM Spd (S3 + Cd). Under this condition, compared with the single cadmium treatment (S0), the seed germination, seedling growth and biomass accumulation under this treatment condition were all significantly improved, and all the indicators (germination potential, germination rate, root length, shoot length, fresh weight and dry weight) were significantly higher, which is consistent with the research results of Gong et al. [18], who reported that exogenous Spd exerts a more pronounced growth-regulating effect under severe stress conditions. Additionally, Naz et al. [36] observed that the application of 0.1 mM Spd under Cr^6+^ stress significantly enhanced plant height, shoot fresh weight, and leaf area, underscoring the involvement of polyamines in plant growth regulation and stress response [37]. Thus, compared to Cd alone, the combined Cd and Spd treatment markedly reduced growth inhibition. However, the promoting effect diminished when Spd concentration increased to 0.5 mM. It is noteworthy that some studies have reported contrasting results. For instance, Recalde et al. [38] found that Spd can act as a signaling molecule to stimulate the production of H_2_O_2_ and NO in wheat under Cd stress, thereby inhibiting root and stem elongation. These findings suggest that the regulatory effects of exogenous Spd on plant growth may vary depending on species-specific responses.

Cadmium exerts profound effects on plant biochemical processes, particularly on soluble protein content, which plays a crucial role in osmotic regulation, enzyme activity maintenance, and stress signal transduction. Numerous studies have indicated that heavy metal stress often leads to the degradation of soluble proteins [39]. In this study, Cd stress significantly reduced soluble protein content in cucumber leaves without Spd application, whereas the addition of 0.2 mM Spd resulted in the highest protein levels (Figure 2 and Figure 3B). This may be attributed to Spd-mediated regulation of proline metabolism, which enhances osmotic protection capacity [39], a finding consistent with Lou et al. [40], who reported that 20 μM Spd significantly increased total protein content in alfalfa under salt stress. Moreover, the disruption of reactive oxygen species (ROS) homeostasis under Cd stress can lead to oxidative damage, as excessive ROS such as ·O_2_^−^ and H_2_O_2_ accumulate and trigger lipid peroxidation. Malondialdehyde (MDA), a byproduct of lipid peroxidation, serves as a key indicator of membrane damage and stress intensity. In this study (Figure 3A), MDA levels in Cd-stressed cucumber leaves without Spd were significantly elevated, aligning with observed reductions in biomass and soluble protein content. However, the addition of 0.2 mM Spd significantly reduced MDA accumulation, suggesting that exogenous Spd mitigates Cd-induced membrane damage and enhances plant tolerance to heavy metal stress. This result is consistent with prior studies [41]. However, at 0.5 mM Spd, the protective effect weakened, possibly due to Spd-induced auto-oxidation or interference with other metabolic pathways [38]. Liu et al. [42] similarly reported that Spd application under As^5+^ stress reduced MDA levels in rice seedlings and roots while effectively scavenging ROS such as ·O_2_^−^, H_2_O_2_, and ·OH.

In addition to inducing oxidative stress, Cd also activates endogenous plant defense mechanisms, among which the antioxidant system is the most extensively studied. This system primarily involves changes in antioxidant enzyme activities and the regulation of non-enzymatic antioxidants. The enzymatic antioxidant system, composed of SOD, POD, CAT, and APX, plays a central role in scavenging ROS and peroxides [43,44]. Research has demonstrated that exogenous Spd can modulate the oxidative status of stressed plants by enhancing antioxidant enzyme activities and reducing ROS accumulation [45]. In this study, Cd stress significantly reduced the activities of CAT, POD, SOD, and APX in cucumber seedlings (Figure 4), potentially due to the promotion of toxic O_2_^−^ generation within cell walls [46]. Exogenous Spd at varying concentrations influenced all four antioxidant enzymes. Compared to the Cd-only treatment, 0.2 mM Spd induced the highest enzyme activities, with a trend of initial increase followed by decrease under higher Spd concentrations. This dual effect may be attributed to two mechanisms: (1) Spd promotes protein synthesis, increasing enzyme abundance; (2) Spd directly binds to enzyme molecules, stabilizing their structure and function, thereby enhancing catalytic efficiency [21].

When plants are exposed to environments contaminated with excessive cadmium (Cd), they inevitably absorb and accumulate this non-essential heavy metal. Due to variations among plant species and cultivars, the patterns of Cd uptake and distribution within plant tissues differ. For example, under high Cd exposure, the distribution of Cd in *Arundo donax* L. follows the order root > stem > leaf [47]. This study found (Table 2) that under Cd stress, Cd accumulation in cucumber seedlings is predominantly localized in the roots, with lower concentrations observed in tender leaves (root > tender leaf), consistent with the findings of Shaheen et al. [42]. Furthermore, the results demonstrate that Cd stress significantly increases Cd content across all plant tissues. In contrast, the application of exogenous Spd at varying concentrations significantly reduces Cd accumulation in both roots and tender leaves, with the most pronounced effect observed at 0.2 mM Spd. This finding aligns with the study by Liu Shujin et al. [48], who reported that Spd application reduces Cd content in rice grains and enhances plant tolerance to stress. These results suggest that exogenous Spd can mitigate Cd accumulation, thereby lowering the risk of Cd (exceeding safety thresholds) and improving plant resistance to Cd toxicity. Additionally, this study observed (Table 2) that Spd application under Cd stress promotes Cd accumulation in underground tissues. This may be attributed to Spd-induced activation of root-associated metal transporters, which enhance Cd sequestration in roots, while simultaneously suppressing Cd translocation from roots to shoots. Consequently, Cd accumulation in aboveground tissues is reduced. To comprehensively assess the regulatory effects of exogenous Spd on physiological and biochemical responses of cucumber seedlings under Cd stress, multi-dimensional statistical analyses were conducted, including correlation analysis, cluster analysis, principal component analysis (PCA), and comprehensive evaluation using a membership function (Figure 5, Figure 6 and Figure 7; Table 3 and Table 4). These analyses revealed intrinsic relationships among key physiological indicators and identified 0.2 mM Spd as the optimal concentration for alleviating Cd-induced stress. Oxidative damage, indicated by elevated malondialdehyde (MDA) levels, was identified as a key factor suppressing seedling growth, including germination, root elongation, and biomass accumulation. The endogenous antioxidant enzyme system, particularly the synergistic network involving peroxidase (POD), catalase (CAT), superoxide dismutase (SOD), and ascorbate peroxidase (APX), serves as the core defense mechanism against oxidative stress and supports continued growth. A strong positive feedback loop exists between germination, growth, and antioxidant system activation. Membership function analysis clearly identified 0.2 mM Spd as the most effective concentration for alleviating Cd stress and improving the overall physiological status of cucumber seedlings under the experimental conditions. Lower concentrations were insufficiently effective, while higher concentrations diminished the beneficial effects. The cadmium transfer factor (TF) exhibited a relatively independent response compared to other stress and defense indicators, suggesting that Cd translocation may be governed by distinct regulatory mechanisms outside the primary stress response pathways. Therefore, gene-level regulatory mechanisms were further explored in this study.

Numerous studies have demonstrated that plants employ highly complex regulatory mechanisms to modulate Cd uptake and accumulation in response to Cd stress, thereby mitigating cellular damage [49]. Among these mechanisms, plant chelatins (PCs) play a crucial role by chelating Cd^2+^ ions to form stable PC-Cd complexes, which reduce the toxicity of free Cd ions within cells. Plant phytochelatin synthase (PCS) is responsible for catalyzing the synthesis of plant chelatins (PCs), a process that uses glutathione (GSH) as the direct precursor [50]. In this study, compared with normally grown cucumber seedlings, Cd treatment significantly induced the accumulation of GSH in leaves, but its content in roots was not significantly affected. This observation aligns closely with the synergistic enhancement of antioxidant enzymes (POD, CAT, SOD, APX) shown in Figure 4, together forming an integrated defense strategy in cucumber seedlings under Cd stress. On the one hand, the antioxidant enzyme system effectively scavenges reactive oxygen species (ROS), thereby reducing oxidative damage, as indicated by elevated MDA levels; on the other hand, the GSH-PCs pathway sequesters Cd ions, lowering their bioavailability and alleviating direct cytotoxic effects. Therefore, the increase in GSH content serves as a key indicator of the systemic activation of Cd stress defense mechanisms in plants. Exogenous application of Spd was found to significantly enhance GSH biosynthesis in leaves and roots of cucumber seedlings under Cd stress (Figure 8), with a clear concentration-dependent effect. While low concentrations of Spd (0.05–0.1 mM; S1 + Cd, S2 + Cd) led to slight increases in GSH levels, these changes were not statistically significant compared to the Cd-only treatment group (S0). The most pronounced effect was observed at 0.2 mM Spd (S3 + Cd), where GSH levels exhibited a sharp increase, particularly in roots directly exposed to Cd. This suggests that 0.2 mM Spd effectively activates the expression or enzymatic activity of genes involved in GSH de novo synthesis or regeneration. Although GSH levels in high-concentration Spd treatments (0.4–0.5 mM; S4 + Cd, S5 + Cd) remained significantly higher than those in S0, they showed a decreasing trend compared to S3 + Cd. This pattern is consistent with the results of the comprehensive evaluation using the membership function, which ranked the overall performance as S3 + Cd > S4 + Cd > S5 + Cd. These findings imply that excessive Spd concentrations may exceed the optimal metabolic capacity of plants, potentially leading to energy burden, feedback inhibition, or other non-specific effects that reduce the efficacy of GSH synthesis and other beneficial physiological processes, such as antioxidant enzyme activity and biomass accumulation.

In addition, previous studies have reported that the rice variety D62B enhances Cd immobilization and vacuolar sequestration through the up-regulation of *OsGST* and *OsPCS1* genes [51]. Similarly, different forms of selenium (e.g., selenocysteine, sodium selenite, and sodium selenate) have been demonstrated to improve Cd tolerance in tomato through the enhancement of GSHS and PC levels and their corresponding gene expression, thereby increasing Cd detoxification capacity [52]. In Chinese cabbage, BcGR1.1 contributes to copper stress tolerance by enhancing GR activity, antioxidant enzyme function, and ascorbate (AsA) utilization, which collectively improve ROS scavenging efficiency [53]. In this study, Cd stress (S0) obviously induced the expression of three key genes—*CsGR*, *CsGSHS*, and *CsPCS1*. The up-regulation of CsGR indicates that Cd-induced ROS overproduction leads to the accumulation of oxidized glutathione (GSSG). The strong induction of CsGR represents a core adaptive response in plants to maintain the intracellular redox balance (GSH/GSSG), ensuring a sufficient supply of reduced GSH for both antioxidant defense and as a precursor for PC synthesis (Figure 9). The up-regulation of *CsPCS1* is a well-established molecular signature of plant responses to heavy metal stress, particularly Cd. PCS1 catalyzes the synthesis of phytochelatins from GSH, which efficiently chelate Cd^2+^ in the cytoplasm, forming low-toxicity complexes that are subsequently transported into vacuoles for compartmentalized storage—a key mechanism for alleviating Cd toxicity. These findings confirm that cucumber seedlings activate the PC synthesis pathway as a core strategy to counteract Cd stress. In contrast, the induction of CsGSHS, the key enzyme gene involved in GSH biosynthesis, was relatively weak and not statistically significant under Cd stress, suggesting that under basal stress conditions, GSH accumulation may primarily rely on GR-mediated recycling of GSSG rather than a substantial enhancement of de novo synthesis.

Exogenous Spd exerts a distinct and differential regulatory effect on genes involved in the GSH-PCs pathway. Under exogenous Spd treatments (0.05–0.5 mM), the expression level of *CsGR* exhibited only minor fluctuations compared to the S0 group (Cd stress alone), with the highest value observed in the S3 + Cd group, although not significantly higher than S0. Nevertheless, CsGR expression remained significantly elevated compared to the control (CK). In contrast to *CsGR*, the expression of *CsGSHS* showed a strong and concentration-dependent response to Spd application. Low concentrations of Spd (S1 + Cd, S2 + Cd) significantly induced *CsGSHS* expression, indicating the activation of de novo GSH synthesis. The expression of *CsGSHS* reached its maximum at the medium Spd concentration of 0.2 mM (S3 + Cd), providing the most direct and critical molecular explanation for the sharp increase in leaf GSH content observed under this treatment. Spd significantly enhances the expression of key GSH biosynthetic enzymes, thereby greatly promoting the plant′s capacity for GSH de novo synthesis. At high Spd concentrations (S4 + Cd, S5 + Cd), *CsGSHS* expression decreased compared to S3 + Cd, although it remained above or comparable to S0 levels. This trend aligns with the observed decline in GSH content under high Spd concentrations (Figure 9), further supporting the existence of an “optimal concentration window” for Spd action, with 0.2 mM being the most effective. The expression pattern of *CsPCS1* clearly illustrates how Spd enhances phytochelatin (PC)-mediated Cd detoxification. At low Spd concentrations (S1 + Cd, S2 + Cd), the induction of *CsPCS1* was relatively weak, suggesting that the bottleneck in PC synthesis at this stage may lie in the limited availability of GSH, the precursor molecule. However, at 0.2 mM Spd (S3 + Cd), CsPCS1 expression was markedly upregulated, indicating that this concentration not only strongly promotes GSH synthesis but also synergistically activates the expression of *CsPCS1*, the key enzyme gene responsible for PC synthesis. The combination of abundant GSH (substrate) and highly active PCS1 (catalyst) synergistically drives the efficient synthesis of PCs. This molecular mechanism perfectly explains the previously observed key phenomenon: under S3 + Cd treatment, leaf GSH content reached its peak while Cd accumulation in the aboveground tissues was the lowest. Efficient PC synthesis significantly enhances Cd chelation and compartmentalization within vacuoles, thereby reducing Cd translocation and accumulation in the shoot and effectively mitigating its toxic effects. At higher Spd concentrations (S4 + Cd, S5 + Cd), *CsPCS1* expression showed minor fluctuations but did not reach the level observed in S3 + Cd, which is consistent with the overall decline in physiological performance observed in the comprehensive evaluation. These findings further confirm that excessive Spd application may exceed the optimal metabolic threshold, leading to diminished efficacy.

## 5. Conclusions

In summary, this study demonstrates that the exogenous application of 0.2 mM Spd effectively mitigates cadmium (Cd) toxicity in cucumber seedlings through a dual defense mechanism. The first mechanism involves active detoxification: Spd specifically activates the CsGSHS-CsPCS1-GSH-PC molecular cascade, promoting the rapid synthesis of glutathione (GSH) and its subsequent conversion into phytochelatins (PCs). This cascade facilitates the efficient chelation and sequestration of Cd within the roots, thereby significantly reducing its translocation to the aboveground tissues. The second mechanism is synergistic antioxidant protection: Spd enhances endogenous GSH levels and maintains its reduced state via up-regulation of glutathione reductase (GR). This, in turn, synergistically interacts with the existing antioxidant enzyme system—including POD, SOD, CAT, and APX—to effectively scavenge reactive oxygen species (ROS) induced by Cd stress, thereby alleviating oxidative damage and lipid peroxidation. The coordinated action of these two mechanisms leads to significant improvements in key physiological processes, including seed germination, plant growth, and biomass accumulation. As a result, cucumber seedlings are able to maintain an optimal overall physiological state under Cd stress, as evidenced by the highest comprehensive performance index (*D* value) observed in this treatment group.

## Figures and Tables

**Table 1:** Effects of Exogenous Spd on Germination and Growth of Cucumber Seeds under Cd stress.

	Treat	Germination Rate	Germination Potential	Root Length/cm	Length of the Young Sprout/cm	Fresh Weight/g	Dry Weight/g
CK	Cd (0 mg·L^−1^) ± 0 mM Spe	0.744 ± 0.019 cd	0.911 ± 0.019 bc	7.243 ± 0.084 d	6.243 ± 0.035 e	2.212 ± 0.004 c	0.190 ± 0.009 abc
S1	0.05 mM Spd	0.744 ± 0.019 cd	0.911 ± 0.019 bc	7.580 ± 0.130 c	6.463 ± 0.038 cd	2.274 ± 0.087 bc	0.193 ± 0.006 abc
S2	0.1 mM Spd	0.833 ± 0.019 ab	0.956 ± 0.019 a	8.350 ± 0.122 a	6.743 ± 0.025 b	2.620 ± 0.228 a	0.202 ± 0.014 a
S3	0.2 mM Spd	0.756 ± 0.019 c	0.933 ± 0.019 ab	7.810 ± 0.061 b	6.583 ± 0.012 c	2.390 ± 0.152 b	0.195 ± 0.010 ab
S4	0.4 mM Spd	0.711 ± 0.019 de	0.900 ± 0.033 bcd	7.590 ± 0.053 c	6.327 ± 0.015 e	1.893 ± 0.007 ef	0.183 ± 0.012 bcd
S5	0.5 mM Spd	0.722 ± 0.019 cd	0.88 ± 0.019 cd	5.963 ± 0.068 e	5.923 ± 0.042 f	1.744 ± 0.027 g	0.173 ± 0.004 d
S0	Cd (10 mg·L^−1^) ± 0 mM Spe	0.678 ± 0.019 e	0.800 ± 0.033 f	5.080 ± 0.035 f	5.020 ± 0.104 h	1.757 ± 0.013 fg	0.180 ± 0.002 cd
S1 + Cd	Cd (10 mg·L^−1^) ± 0.05 mM Spe	0.711 ± 0.019 de	0.844 ± 0.019 e	5.153 ± 0.064 f	5.520 ± 0.030 g	1.877 ± 0.024 efg	0.188 ± 0.001 abc
S2 + Cd	Cd (10 mg·L^−1^) ± 0.1 mM Spe	0.744 ± 0.019 cd	0.867 ± 0.019 de	7.730 ± 0.191 b	7.060 ± 0.044 a	2.040 ± 0.048 d	0.192 ± 0.004 abc
S3 + Cd	Cd (10 mg·L^−1^) ± 0.2 mM Spe	0.844 ± 0.019 a	0.956 ± 0.019 a	8.243 ± 0.040 a	7.210 ± 0.010 a	2.198 ± 0.006 c	0.202 ± 0.010 a
S4 + Cd	Cd (10 mg·L^−1^) ± 0.4 mM Spe	0.800 ± 0.033 b	0.911 ± 0.019 bc	7.803 ± 0.023 b	6.463 ± 0.093 cd	2.227 ± 0.021c	0.194 ± 0.002 abc
S5 + Cd	Cd (10 mg·L^−1^) ± 0.5 mM Spe	0.811 ± 0.019 ab	0.922 ± 0.019 abc	7.490 ± 0.052 c	6.363 ± 0.051 de	2.001 ± 0.121 de	0.191 ± 0.004 abc

Note: The values represent the mean ± SD, with three replicates; different lowercase letters indicate significant differences (*p* < 0.05).

**Table 2:** The effect of exogenous Spd on cadmium content in roots and young leaves of cucumber seedlings under Cd stress.

Treatment Group	Cd Content/(mg·kg^−1^)		Transfer Coefficient
	Cucumber Leaves	Cucumber Root System	
CK (0 mg·L^−1^ Cd + 0 mM Spe)	_	_	_
S0 (10 mg·L^−1^ Cd + 0 mM Spd)	227.71 ± 18.57 a	1261.06 ± 27.69 a	0.18 ± 0.012 a
S1 + Cd (10 mg·L^−1^ Cd + 0.05 mM Spe)	190.88 ± 7.73 b	1080.06 ± 35.02 b	0.177 ± 0.005 a
S2 + Cd (10 mg·L^−1^ Cd + 0.1 mM Spe)	140.27 ± 12.31 c	807.9 ± 40.28 c	0.173 ± 0.009 ab
S3 + Cd (10 mg·L^−1^ Cd + 0.2 mM Spe)	88.45 ± 6.87 e	597.5 ± 24.73 e	0.148 ± 0.008 c
S4 + Cd (10 mg·L^−1^ Cd + 0.4 mM Spe)	109.27 ± 7.25 de	727.69 ± 40.3 d	0.151 ± 0.015 bc
S5 + Cd (10 mg·L^−1^ Cd + 0.5 mM Spe)	126.36 ± 13.84 cd	839.21 ± 40.76 c	0.151 ± 0.021 bc

Note: The different lowercase letters in a column indicate significant differences among treatments at *p* < 0.05 levels. “_” indicates the absence of detectable cadmium (Cd) content in plant leaves and roots under blank control conditions.

**Table 3:** Principal component analysis of all indicators, as well as eigenvalues, contribution rates and cumulative contribution rates.

	Principle Component		
Index	PC	PC	PC
GP	0.28015	0.03743	−0.32612
SP	0.26329	−0.15354	−0.34143
RL	0.29819	−0.11584	0.01789
SG	0.28438	−0.09624	0.38088
FW	0.25114	−0.2859	−0.19881
DW	0.24441	−0.01558	0.40581
SP*	0.2698	−0.01177	0.1837
MDA	−0.29328	0.0515	−8.58 × 10^−6^
POD	0.30365	0.05793	−0.0434
CAT	0.29079	0.16558	0.12995
SOD	0.27199	−0.22769	−0.00681
APX	0.28728	0.09384	0.24409
Cd-L	0.1826	0.47004	−0.31241
Cd-R	0.1826	0.47004	−0.31241
TF	−0.02886	0.57817	0.3466
Eigenvalue	10.16796	2.57388	0.68186
Contribution rate	67.79%	17.16%	4.55%
Cumulative contribution rate	67.79%	84.95%	89.49%

**Table 4:** Integrated scores of each component calculated based on membership functions.

Treatment	PAC1	PAC2	PAC3	X1	X2	X3	Dvalue	Comprehensive Sorting
CK	−0.512	−0.198	0.305	0.476	0.484	1.000	0.532	2
S0	−2.105	−1.876	−0.450	0.000	0.000	0.000	0.000	7
S1 + Cd	−1.320	−0.754	−0.120	0.147	0.290	0.412	0.192	6
S2 + Cd	1.856	0.432	−0.305	0.742	0.595	0.179	0.648	3
S3 + Cd	3.241	1.985	0.112	1.000	1.000	0.742	0.939	1
S4 + Cd	2.674	1.203	−0.208	0.895	0.795	0.304	0.791	4
S5 + Cd	1.987	0.618	−0.450	0.765	0.643	0.000	0.612	5
Weighting				w1 = 0.757	w2 = 0.192	w3 = 0.051		

## Data Availability

The original data and experimental protocols can be made public to the scientific community for replication.

## Supplementary Materials

The following supporting information can be downloaded at: https://www.mdpi.com/article/10.3390/toxics13100822/s1, Table S1. The primers of RT-qRCR used in this study.
