# Supplementary material for: Exogenous Spermidine Induces Cadmium Stress Tolerance in Cucumber Seedlings by Promoting Plant Growth and Defense System
Source: Toxics. 2025 Sep 26;13(10):822. doi: 10.3390/toxics13100822 (PMC12567920; doi:10.3390/toxics13100822)
Supplement: Supplementary file 1 [file toxics-13-00822-s001.zip › toxics-3873067-supplementary.pdf]

Table S1 The primers of RT-qRCR used in this study

**Analysis**	**Primer name**	**Sequence(5′-3′)**	**Tm/℃**	**Amplicon size/bp**
CsGR for qRT-RCR in transgenic cucumber	CsGR-F<br>CsGR-R	TGCTGTTTGCCGCAGACTTAG<br>AAAGTCGTAGTGTTCCTCCGCTTC	60<br>60	109
CsGSHS for qRT-RCR in transgenic cucumber	CsGSHS-F<br>CsGSHS-R	TGTTGGTGACAAGTCTGTGAAGAG<br>GGCAATTGGTGAATGAGACAAACC	58<br>58	70
CsPCS1 for qRT-RCR in transgenic cucumber	CsPCS1-F<br>CsPCS1-R	TTCTTCCTTCTCCTCCAGCAATCG<br>CAGCTTCTACGAAGAGTTGCTTCC	58<br>58	66
Cucumber *CsActin* gene for and qRT-PCR	Actin-F<br>Actin-R	TCGTGCTGGATTCTGGTG<br>GGCAGTGGTGGTGAACAT	60<br>60	161
